# Atypical and Late-Developed Sinus Graft Complications Following Maxillary Sinus Augmentation: Successful Management with Guided Bone Regeneration

**DOI:** 10.3390/medicina60081246

**Published:** 2024-07-31

**Authors:** Won-Bae Park, Kenechi P. Okany, Wonhee Park, Ji-Young Han, Hyun-Chang Lim, Philip Kang

**Affiliations:** 1 Department of Periodontology, School of Dentistry, Kyung Hee University; Seoul 02447, Republic of Korea; wbpdds@naver.com; 2 Private Practice in Periodontics and Implant Dentistry, Seoul 02771, Republic of Korea; 3 Division of Periodontics, Section of Oral, Diagnostic and Rehabilitation Sciences Columbia University College of Dental Medicine, #PH7E-110, 630 W. 168 St., New York, NY 10032, USA; kpo2110@cumc.columbia.edu; 4 Department of Dentistry, Division of Dentistry, College of Medicine, Hanyang University, Gyeongchun-ro, Guri-si 11923, Republic of Korea; whpark@hanyang.ac.kr; 5 Department of Periodontology, Division of Dentistry, College of Medicine, Hanyang University, 222-1 Wangsimni-ro, Seongdong-gu, Seoul 04763, Republic of Korea; hjyperio@hanyang.ac.kr; 6 Department of Periodontology, Periodontal-Implant Clinical Research Institute, School of Dentistry, Kyunghee Daero 23, Dongdaemoon-gu, Seoul 02447, Republic of Korea

**Keywords:** complication, dental implant, guided bone regeneration, maxillary sinus augmentation, sinus graft complication

## Abstract

Complications that occur after maxillary sinus floor augmentation (MSA) can be divided into early and late complications. Early complication is a side effect that occurs during the MSA procedure or during the initial healing period. Usually, late complication refers to a side effect that occurs after 3 weeks of MSA. However, in the longer term, there are cases that occur during the follow-up period after the prosthesis is delivered, and most of them present with peri-implantitis. In the present two cases, sinus graft complications occurred 1–2 years after prosthesis delivery but were independent of peri-implantitis and had atypical features showing asymptomatic results. Although the route of the infection source is unclear, the lesions were presumed to be caused by slow and delayed inflammation of oral bacteria infiltrating the bone graft area of the maxillary sinus. Within the limitations of present case reports, bone defects were successfully managed with a guided bone regeneration (GBR) procedure that included thorough defect degranulation, surface decontamination of exposed implant, and regrafting. Periodic monitoring of radiographic images is required for the detection of unusual sinus graft complications in sinus-augmented sites.

## 1. Introduction

Maxillary sinus augmentation (MSA) enables successful implant placement when maxillary posterior ridges are severely atrophied, and maxillary sinuses are pneumatized [1,2,3]. MSA has a long-term survival rate of 95% at 10 years and 85% at 20 years and is evaluated as a procedure with clinical predictability [2]. Therefore, the number of procedures performed is increasing, and in line with this, the improvement in technique, the introduction of various sinus elevation instruments, and the development of biomaterials have continued.

Autogenous bone is the gold standard for bone graft materials in MSA. However, it has been replaced by allo-, xeno-, and synthetic bone grafts, and in some cases, it is used by mixing with autogenous bone in an appropriate ratio [4,5]. A high success rate was reported for maxillary sinus bone grafts regardless of the type of bone graft material [1,2,3]. It is common to cover the lateral window site with a barrier membrane or to reposition the lateral window for the success of guided bone regeneration (GBR) [6].

The most common complications that occur during MSA include perforation of the sinus membrane, and complications associated with sinus membrane perforation include leakage of bone graft particles, sinus membrane thickening, ostium plugging, dysventilation due to impaired mucociliary clearance, early sinus graft infection, maxillary sinusitis, and postoperative maxillary cysts [7,8,9,10,11,12]. However, it is a general opinion that complications are rare if the perforated sinus membrane is well repaired. Recently, there has also been a study that showed no clinical and radiological differences between the perforation group and the non-perforation group in the lateral window approach [13]. The harmfulness of sinus membrane perforation is controversial.

Lesions showing radiolucent or heterogenous images within the maxillary sinus bone graft have been reported to include sinus graft infection, grafting void, and surgical ciliated cyst [12,14,15,16]. Sinus graft infection occurs early or late, usually with intraoral and sinonasal symptoms. Asymptomatic sinus graft infection is uncommon. A surgical ciliated cyst, also called a postoperative maxillary cyst, occurs after radical sinus surgery and is caused by the inflow of ciliated pseudostratified columnar epithelium during the procedure. Surgical ciliated cysts have been reported to develop even after MSA and are found on radiographs months to years after MSA [17]. A grafting void is another early complication that can occur regardless of sinus membrane perforation and is sometimes confused with a surgical ciliated cyst if the size is large [2].

Late complications that may occur after MSA are mainly caused by advanced peri-implantitis [18]. Sinus graft infection with bone sequestration, oroantral fistula, implant displacement, maxillary sinusitis, and paranasal sinusitis have been reported as complications associated with peri-implantitis at the MSA site [8,18,19]. Because these complications occur in combination, there are many difficulties and variables for successful treatment.

To the author’s knowledge, atypical late sinus graft complications caused by non-peri-implantitis have not reported. In the present two cases, late sinus graft complications caused by non-peri-implantitis will be introduced, and the treatment with GBR will be described.

## 2. Case Presentations

### 2.1. Case 1

**Figure 1:**
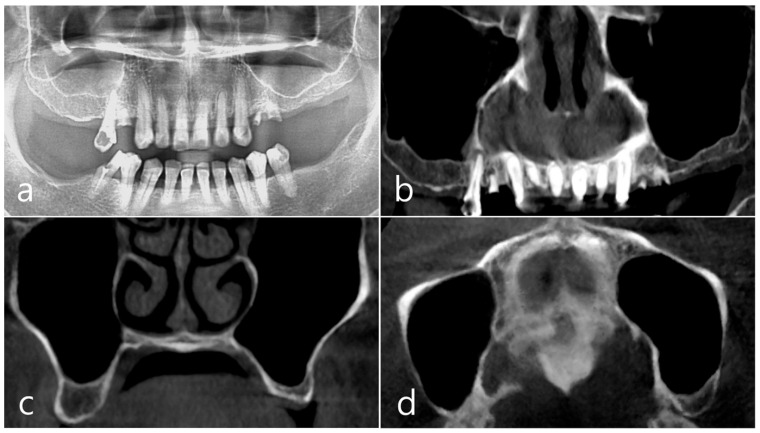
Case 1. (**a**) In panoramic radiography, resorption of residual alveolar ridge and pneumatization of the maxillary sinus were observed in the left and right maxillary posterior region; (**b**–**d**) In preoperative CBCT’s panoramic, coronal, and axial images, there were no abnormal findings of the maxillary sinus and no sinus membrane thickening.

This patient, a 67-year-old female non-smoker, reported no systemic disease that could interfere with the operation. The patient visited the clinic for implant placement for the missing teeth. Panoramic radiography and CBCT were taken. On panoramic radiography, resorption of residual alveolar ridge and pneumatization of the maxillary sinus were observed in the left and right posterior region. Since the amount of residual alveolar bone was 4–6 mm in the maxillary right posterior region, a transcrestal sinus lift was planned (Figure 1a). In the preoperative panoramic, coronal, and axial images of the CBCT scan, abnormal images of the maxillary sinus were not observed, and there was no sinus membrane thickening (Figure 1b–d).

**Figure 2:**
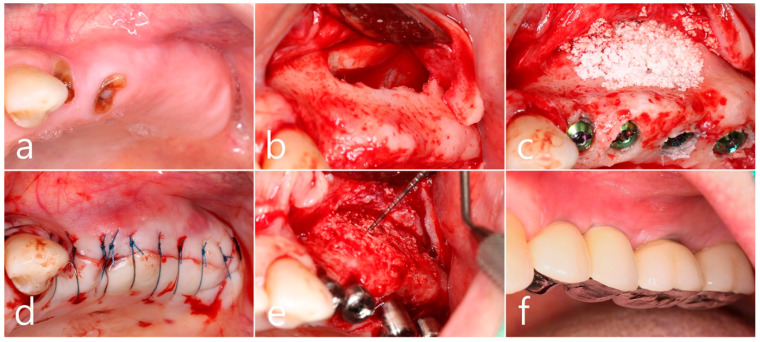
(**a**) Preoperative clinical photograph of the maxillary left posterior region showed root rest of #24 and #25 teeth; (**b**) After the mucoperiosteal flap was reflected, sinus floor elevation was performed. The sinus membrane was not perforated; (**c**) A bone graft substitute was filled in the elevated sinus cavity, and 4 implants were placed subcrestally. The thickness of the buccal bone was sufficiently maintained; (**d**) The lateral window site was covered with a resorbable collagen membrane and then closed with 4-0 nylon; (**e**) Uncovering was performed 6 months after surgery. Resistance was felt when penetrated with a periodontal probe; (**f**) Prosthesis was delivered 2 months later.

In the preoperative clinical picture of the maxillary left posterior region, root rest of #24 and #25 teeth were observed (Figure 2a). Under local anesthesia, the buccal mucoperiosteal flap was reflected, a lateral sinus window was prepared, and the sinus floor was elevated using a sinus elevation instrument (Genoss, Suwon, Republic of Korea). The sinus membrane was not perforated (Figure 2b). Approximately 1.5 cc of Osteon II (Genoss, Suwon, Republic of Korea) was filled in the elevated sinus cavity, and 4 implants (3.8 × 12, 4.3 × 10 Implantium, Dentium, Suwon, Republic of Korea) were placed subcrestally. The thickness of the buccal bone in the ridge crest area was sufficiently maintained (Figure 2c). The lateral window site was covered with a resorbable collagen membrane (Genoss, Suwon, Republic of Korea), and the mucoperiosteal flap was then closed with 4-0 nylon (Figure 2d). Healing was uneventful. Uncovering was performed 6 months after surgery. Resistance was felt when penetrated with a periodontal probe. It was confirmed that bone regeneration was successful. The cover screw was removed, and the healing abutment was inserted. The mucoperiosteal flap was closed (Figure 2e). After 2 months, the final prosthesis was delivered (Figure 2f).

**Figure 3:**
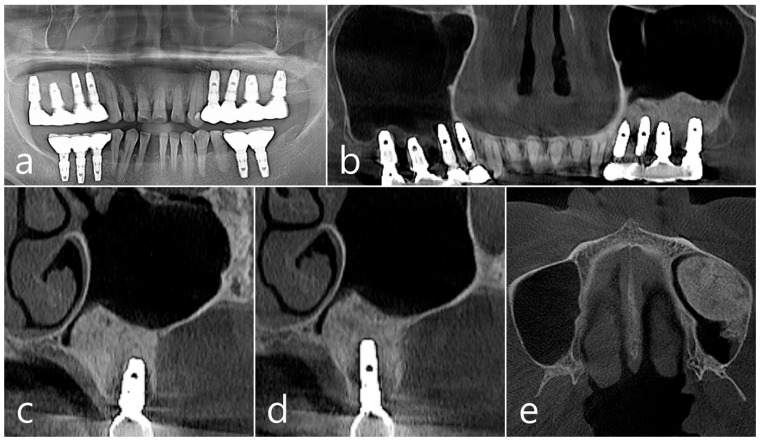
(**a**,**b**) In the panoramic radiography and CBCT images taken after the prosthesis was delivered, no special radiographic image was observed at the left sinus augmented site; (**c**,**d**) In coronal images of CBCT scanned in #26 and #25 implants, a homogenous radiographic density was observed at the sinus augmented site, and there was no thickening of the sinus mucosa; (**e**) A well-augmented sinus graft was observed in CBCT axial image.

**Figure 4:**
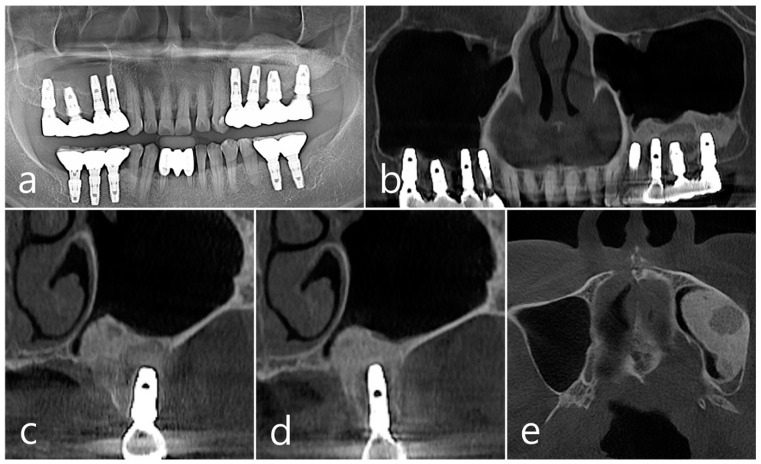
(**a**,**b**) In the panoramic radiography and CBCT images taken 1 year after prosthesis delivery, unusual radiolucent images were observed around implants #25 and #26; (**c**,**d**) Radiolucent images around #26 and #25 implants were observed. The buccal cortical plate was lost. However, the appearance of the sinus graft was well maintained, and sinus mucosal thickness was normal; (**e**) A circular radiolucent finding was observed in the axial image of CBCT.

In the panoramic radiography and CBCT images taken after the prosthesis was delivered, no unusual radiographic image was observed at the bone graft site in the left maxillary sinus (Figure 3a,b). In the coronal images of the CBCT scans in #25 and #26 implants, no specific finding was found at the bone graft site of the maxillary sinus, and there was no thickening of the sinus membrane (Figure 3c,d). A well-consolidated sinus graft was observed on the axial image of CBCT (Figure 3e). The patient was checked up twice a year, and panoramic radiography and CBCT were taken 1 year after prosthesis delivery. Radiolucent images were observed around implants #25 and #26 (Figure 4a–d). A circular radiolucent image was observed in the axial image of CBCT (Figure 4e).

**Figure 5:**
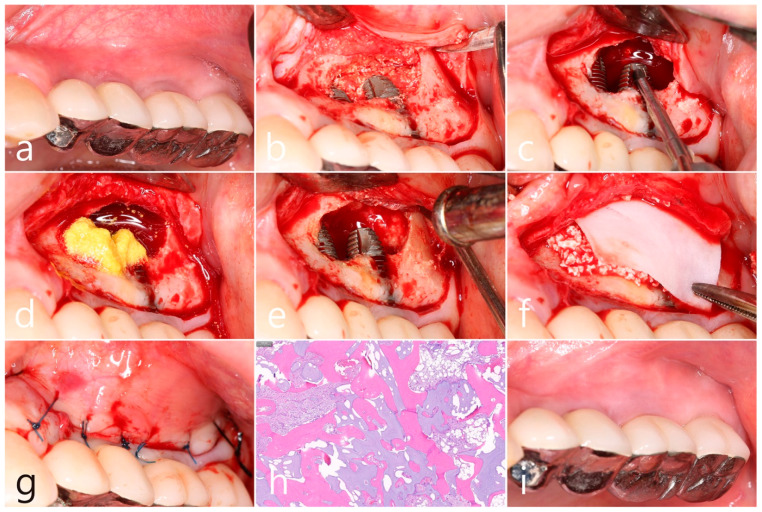
(**a**) In the clinical findings 1 year after prosthesis delivery, there were no clinical symptoms; (**b**) The buccal mucoperiosteal flap was reflected. The periapical bone and facial bone plate of #25 and #26 implant apexes were resorbed, and the implants were exposed; (**c**) Granulation tissue within the bone defect was thoroughly removed, and a titanium brush and titanium curette were used to decontaminate the implant surface; (**d**) Additional detoxification was performed with tetracycline HCl on the exposed implant surface; (**e**) Thorough saline irrigation was performed; (**f**) The bone defect was filled with bone graft substitute and covered with a resorbable collagen membrane; (**g**) The flap was closed with 4-0 nylon; (**h**) Necrotic bone and bone graft particles were scattered throughout the specimen, and inflammatory cell infiltration was also seen (H-E stain). (**i**) Healing was uneventful. No abnormal appearance was observed in the clinical findings 5 years after GBR.

In the clinical findings 1 year after prosthesis delivery, there were no clinical signs such as edema or fistula (Figure 5a). Surgical intervention was decided according to the radiographic images. Under local anesthesia, the buccal mucoperiosteal flap was reflected. The peripheral bone of the #25 and #26 implant apexes was resorbed, and the implant surfaces were exposed. The facial bone of implant #26 was also absorbed, exposing the implant surface (Figure 5b). Inflammatory granulation tissue within the bone defect was thoroughly removed, and a titanium brush and curette were used to decontaminate the implant surface (Figure 5c). Additional detoxification was performed with tetracycline HCl on the exposed implant surface (Figure 5d). Thorough saline irrigation was performed (Figure 5e). The bone defect was filled with Osteon II (Genoss, Suwon, Republic of Korea) and covered with a resorbable collagen membrane (Genoss, Suwon, Republic of Korea) (Figure 5f). The mucoperiosteal flap was closed with 4-0 nylon (Figure 5g). The specimen was fixed in 10% formalin for histopathological examination. Necrotic bone and bone graft particles were scattered throughout the specimen, and inflammatory cell infiltration was also seen. However, no pseudostratified columnar epithelium was found (Figure 5h). Healing was uneventful. No abnormal findings were observed in the clinical findings 5 years after GBR (Figure 5i).

**Figure 6:**
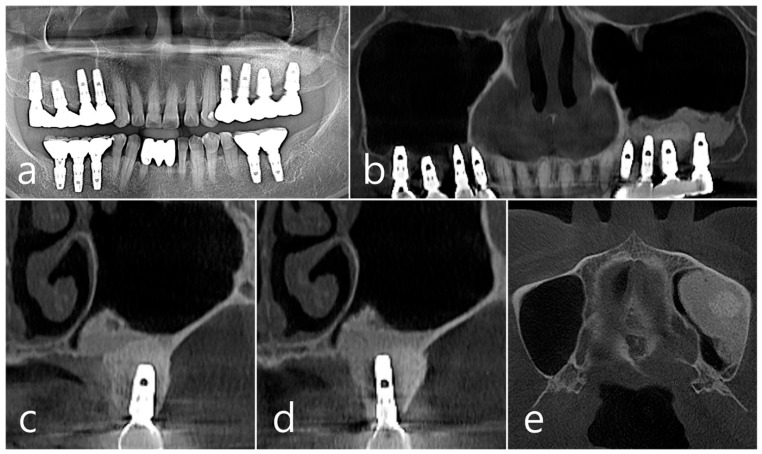
(**a**,**b**) In panoramic radiography and panoramic image of CBCT taken 5 years after GBR, previous radiolucent images were no longer observed; (**c**) Well-consolidated bone graft was observed around the #26 implant; (**d**) Bone formation and corticalization on the buccal side of the #25 implant was well achieved; (**e**) A well-consolidated bone graft was observed in the CBCT axial image.

In panoramic radiography and CBCT panoramic images taken 5 years after GBR, previous radiolucent images were no longer observed (Figure 6a,b). Well-grafted bone was observed around the #26 implant, and it was observed that it was well-integrated into the adjacent native bone. The resorbed facial bone was also reformed (Figure 6c). Bone regeneration and consolidation on the facial side of the #25 implant were well achieved (Figure 6d). A well-consolidated bone graft was observed in the CBCT axial image (Figure 6e).

### 2.2. Case 2

**Figure 7:**
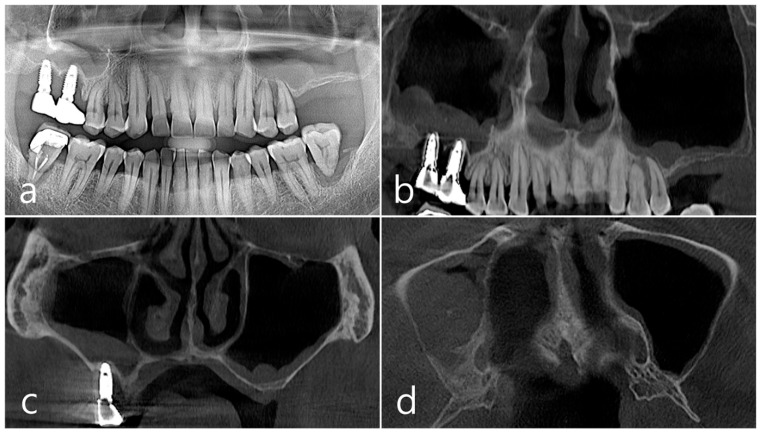
Case 2. (**a**,**b**) On the preoperative panoramic radiography and panoramic image of CBCT, the residual alveolar ridge in the posterior maxilla was severely atrophied and pneumatization of maxillary sinus was severe; (**c**,**d**) In CBCT imaging, mucosal thickening (arrows) ranging from 2 to 15 mm was observed in the sinus floor, depending on the location, but there was no mucosal thickening in the rest of the sinus wall.

This patient was a 58-year-old male smoker who was taking antihypertensive drugs. The patient visited our clinic for implant placement in the upper left posterior region. Teeth #24 and #25 were determined to be extracted due to severe periodontal disease. In panoramic radiography, the residual alveolar bone was severely atrophied, and pneumatization of the maxillary sinus was severe (Figure 7a). In CBCT imaging, membrane thickening was observed in the sinus floor, but there was no membrane thickening in the rest of the sinus wall (Figure 7b–d).

**Figure 8:**
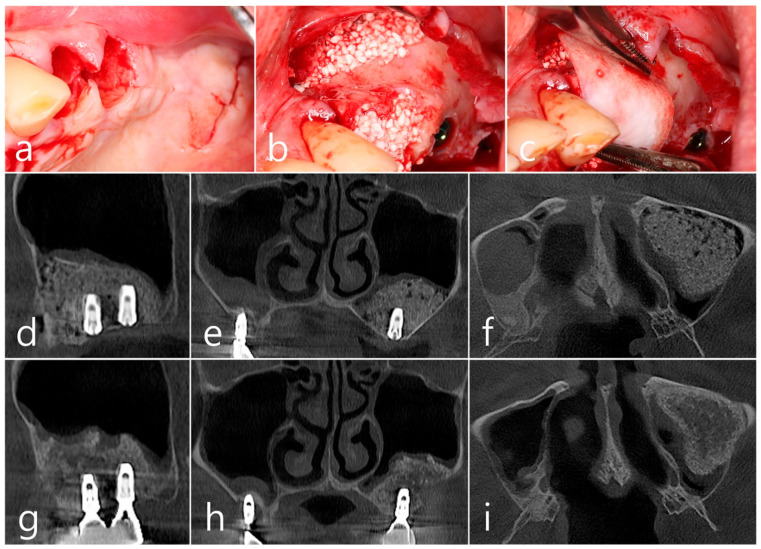
(**a**) Teeth #24 and #25 were extracted, midcrestal and vertical incisions were performed, and mucoperiosteal flaps were reflected; (**b**,**c**) A lateral sinus window was prepared, and sinus floor elevation was performed without membrane perforation. The bone graft substitute was delivered and covered with a resorbable collagen membrane. The flap was closed; (**d**–**f**) In the CBCT image taken immediately after the procedure, a well-filled bone graft substitute without displacement was observed; (**g**–**i**) Extensive bone resorption around #26 implant was observed in CBCT images taken 2 years after prosthesis delivery. The buccal cortical bone plate was lost, and bone resorption was present only within the sinus graft.

Under local anesthesia, teeth #24 and #25 were extracted, midcrestal and vertical incisions were performed, and mucoperiosteal flaps were reflected (Figure 8a). A lateral sinus window was prepared in the buccal sinus wall, and sinus floor elevation was performed without membrane perforation. As a bone graft substitute, 1 cc of Osteon II (Genoss, Suwon, Republic of Korea) hydrated with saline solution was used (Figure 8b), and a resorbable collagen membrane (Genoss, Suwon, Republic of Korea) was covered (Figure 8c). The flap was closed using 4-0 nylon. Healing was uneventful, and there were no clinical symptoms until 6 months after surgery.

In the CBCT image taken immediately after the procedure, a well-filled bone graft substitute without perforation of the sinus membrane was observed (Figure 8d–f). However, extensive bone loss was found in CBCT images taken 2 years after the prosthesis was placed. The facial cortical bone plate was lost, and bone resorption was present only within the sinus graft. The appearance of the sinus graft was not invaded, and the thickness of the sinus mucosa was normal (Figure 8g–i).

**Figure 9:**
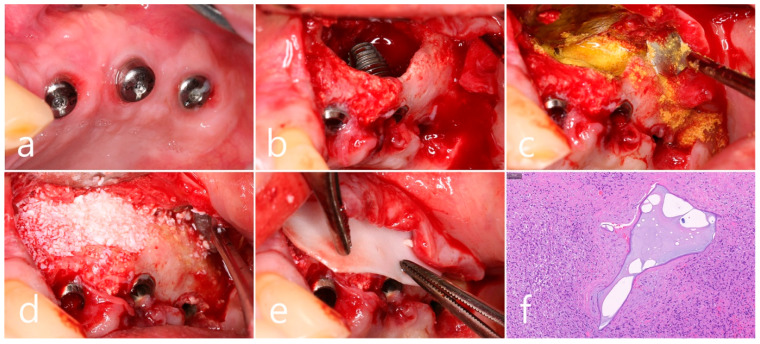
(**a**) Surgical intervention was decided after the prosthesis was retrieved; (**b**) The buccal mucoperiosteal flap was reflected. Thorough debridement was performed on the bone defect in the maxillary sinus bone graft; (**c**) Mechanical decontamination was thoroughly performed using a titanium brush and a titanium curette, and additional detoxification with tetracycline HCl was performed after sufficient saline irrigation; (**d**) A bone graft was performed; (**e**) The bone graft area was covered with a resorbable collagen membrane. The mucoperiosteal flap was closed. Healing was uneventful; (**f**) In the specimen, granulation tissue infiltrated with inflammatory cells, and necrotic bone graft particles were observed (H-E stain).

**Figure 10:**
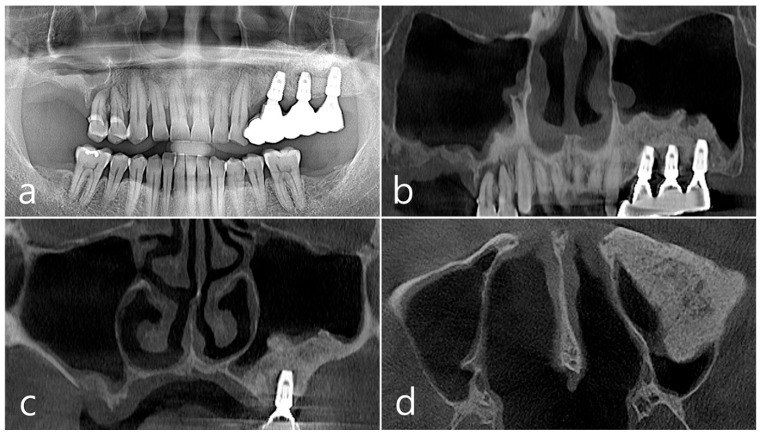
(**a**,**b**) In panoramic radiography and CBCT images taken 2 years after GBR, the sinus graft site of the maxillary sinus was well filled with bone tissue, and no abnormal radiographic images were observed; (**c**,**d**) On coronal and axial images of CBCT, homogeneous findings were observed in the previous bone defect site. Corticalization of the facial bone plate was well achieved.

Surgical intervention was decided after the prosthesis was retrieved (Figure 9a). Under local anesthesia, the buccal mucoperiosteal flap was reflected. A severe bone defect was observed in the sinus bone graft of the #26 implant. Thorough debridement was performed on the bone defect in the maxillary sinus bone graft. There was no difficulty in removing the inflammatory granulation tissue because it bordered the surrounding healthy bone graft (Figure 9b). The exposed implant achieved osseointegration, but the large implant surface was exposed and contaminated. Mechanical decontamination was thoroughly performed using a titanium brush and titanium curette, and additional detoxification with tetracycline HCl was performed (Figure 9c). After sufficient saline irrigation again, a bone graft (Osteon II, Genoss, Suwon, Republic of Korea) was performed (Figure 9d). The bone graft area was covered with a resorbable collagen membrane (Genoss, Suwon, Republic of Korea) (Figure 9e). The mucoperiosteal flap was closed with 4-0 nylon. Healing was uneventful, and the prosthesis was re-inserted again 1 month after GBR. The specimen was fixed in 10% formalin for histopathological examination. In the specimen, granulation tissue infiltrated with inflammatory cells, and necrotic bone graft particles were observed (Figure 9f). The patient was followed up once every 6 months, and panoramic radiography and CBCT were taken 2 years after GBR (Figure 10a,b). The bony defect was filled with bone tissue, and no abnormal radiographic images were observed. Corticalization of the buccal bone was well achieved (Figure 10c,d).

## 3. Results

In two cases, the sinus-augmented site was infected due to the inflow of bacteria through an unknown route, and consolidation of the sinus graft eventually failed. The lesions were found on radiographic images taken during regular follow-up checks after the prosthesis was delivered, and there were no signs or symptoms in the patients. This was a peculiarity different from the common early or late sinus graft infection. Contaminated implants with osseointegration were not removed and were treated well with GBR procedures, including defect degranulation, surface decontamination, and regrafting.

## 4. Discussion

In present case reports, despite the lack of microbiological cultures, two cases of atypical late-developed asymptomatic sinus graft complications, which were presumed to be delayed expression of sinus graft infection without invasion of the maxillary sinus, were introduced, and GBR procedure, including defect degranulation, mechanical/chemical decontamination, and regrafting were performed. The results showed GBR was successfully achieved clinically and radiologically.

Early sinus graft infection usually develops acutely within 3 weeks after MSA [20]. It is accompanied by symptoms such as pain, buccal swelling, fistula formation, and headache. In addition, flap dehiscence, oroantral communication, loss of graft material, and implant displacement may also occur [20]. Occasionally, maxillary sinusitis occurs concomitantly, so consultation with an otolaryngologist may be required, or functional endoscopic sinus surgery may be performed [19]. If sinus graft infection does not invade the sinus membrane too much, antibiotic therapy, incision and drainage, removal of the implant, and bone regrafting are good treatment choices [14,15]. Early and appropriate intervention can preserve maximal bone grafts and enable implant survival [15].

Testori et al. (2023) classified side effects that occurred 3 weeks after MSA as late complications and introduced late sinus graft infection, loss of osseointegration, and implant displacement as late complications [20]. Late complications, such as late sinus graft infection due to peri-implantitis developed during long-term follow-up, have also been reported [8,18,21]. Park et al. reported cases of late sinus graft infection due to peri-implantitis accompanied by oroantral fistula, implant displacement, graft sequestration, and paranasal sinusitis. In addition, late sinus graft infection caused by peri-implantitis is not limited to sinus bone grafting but is accompanied by sinus membrane thickening, sinus opacification, and paranasal sinusitis [8,18]. Contrary to their reports, present cases were detected without clinical symptoms 1–2 years after prosthesis delivery. Since the bone-to-implant contact in the crestal portion of the implant was not violated and sinus membrane thickening was not accompanied, it is similar to retrograde or lateral peri-implantitis occurring within the bone graft area of the maxillary sinus [22,23]. Therefore, it has atypical features that are different from those of the previously reported late sinus graft infection in its onset timing and pattern. In addition, the atypical characteristics of the cases may have been due to non-infectious causes, which may present differently than other reported cases caused by infections.

The presented late sinus graft complication was different from a grafting void in terms of the time of occurrence. Grafting voids occur early after MSA, and their size decreases over time, and eventually, only traces are visible on radiographic images [2]. However, cases where the grafting void was abnormally large and looked like a surgical ciliated cyst have been reported [16]. Case 1 showed radiographic findings similar to those of surgical ciliated cysts. The surgical ciliated cyst in the maxillary sinus bone graft is caused by the inflow of ciliated epithelium through the perforation of the sinus membrane [24]. However, histologically, the surgical ciliated cyst showed pseudostratified ciliated columnar epithelium, but in these cases, this cell was not identified, and only inflammatory cells and necrotic graft particles were observed. Also, there was no perforation of the sinus membrane during sinus floor elevation.

The cause and pathway of late sinus graft infection are difficult to explain with certainty, and some complications are caused by non-infectious reasons as well. However, bacteria can penetrate the sinus graft due to salivary contamination or wound exposure during the uncovering procedure or delivery of the bone graft material. It is presumed that this infection proceeds very slowly and chronically so that the patient does not show any clinical symptoms and it does not affect the adjacent sinus membrane. In Case 1, no suspicious image was observed in the bone graft area of the maxillary sinus in the CBCT scan taken after the prosthesis was delivered, and in Case 2, graft complications were confirmed on CBCT images taken 2 years after prosthesis delivery. Even with the implant exposed to the sinus graft infection, osseointegration was achieved and the chewing function was maintained. Therefore, the explanation of the implant was not considered. Implants involved in infection are explanted when the contaminated area is large, instrument access is difficult, and osseointegration is not achieved.

The present late sinus graft complication was confined to the sinus graft and did not affect the thickness of the adjacent maxillary sinus mucosa. Therefore, it was treated in a similar way to an early sinus graft infection and retrograde peri-implantitis [14,15,25,26,27]. In the treatment of an early sinus graft infection, if the primary stability of the implant is well obtained, successful treatment results can be obtained with defect degranulation, surface decontamination, and regrafting. Treatment of retrograde peri-implantitis includes apical implant resection [25,26,27] and mechanical and chemical decontamination of the exposed implant surface [15,28,29]. None of the treatment methods for contaminated implant surfaces have been established and proven to be appropriate and effective [30]. Therefore, combined therapy using various methods is widely applied in clinical practice [31]. As in presented two cases, the cases in which a titanium brush, titanium curette, and tetracycline HCl were successfully used as surface decontamination methods were (1) implants exposed in the maxillary sinus [32], (2) implants exposed to postoperative maxillary cysts [33], (3) implant exposed to early sinus graft infection [14,15], (4) implant exposed to lateral peri-implantitis [23]. These reports pointed out that the mechanical and chemical decontamination methods used in present cases are effective for implant surface contamination due to non-peri-implantitis.

In addition, there is a difference in effectiveness between the treatment of the implant surface contaminated at the sinus-augmented site and the treatment of the implant surface contaminated by peri-implantitis. Contamination by peri-implantitis inevitably reduces efficiency because of biofilm formation by various bacteria and contaminants accumulated [34,35,36]. Therefore, even regenerative procedures targeting decontaminated implant surfaces have a low success rate. In contrast, a sinus graft infection is the same in terms of bacterial infection, but since it occurs in a limited space and in a shorter period than peri-implantitis, the degree of contamination is relatively low, and the decontamination effect increases. As in the presented cases, it was judged that GBR performed after defect degranulation and mechanical and chemical decontamination could achieve successful results. If the contaminated surface of the implant body is wide, replacing it with a new implant is also a good option, but the patient’s treatment period and the period of chewing discomfort are prolonged, and the complaint is severe.

Non-resorbable polytetrafluoroethylene (PTFE) and resorbable collagen membranes are popular barrier membranes used for peri-implant defects [37,38,39]. Consent from the patient is difficult to obtain due to the need to remove the non-resorbable membrane. Resorbable collagen membranes have difficulties in gaining regenerative space due to limitations in physical properties, but it has been confirmed that mechanical stability is increased through the cross-linking process, and additionally, osteogenic potential is present [39,40]. Therefore, the resorbable collagen membrane plays a sufficient role as an alternative to the non-resorbable PTFE membrane. The key is to achieve re-osseointegration between the regrafted bone and the treated implant surface. Validated histological results for this have not yet been reported. If there are no clinical symptoms and no abnormal images are found on radiographs, it is an acceptable state.

Even if asymptomatic after MSA, regular 3-D radiographic images are required. Clinicians should be aware of late sinus graft complications and consider early surgical intervention if detected. The disadvantage of this case report is that the number of cases is small, so there is a limit to obtaining results based on evidence. Hopefully, more research will be undertaken in the future.

## 5. Conclusions

Within the limitations of the case reports, successful results were achieved with GBR techniques, including defect debridement, surface decontamination of exposed implants, and regrafting in an atypical late-developed sinus graft complication.

## Data Availability

The data presented in this study are available on request from the corresponding author due to privacy.
